# Occupational health, integrative and complementary practices in primary care, and the Covid-19 pandemic

**DOI:** 10.1590/1980-220X-REEUSP-2021-0362

**Published:** 2022-03-28

**Authors:** Erika Cardozo Pereira, Marlene Pereira da Rocha, Lissandra Zanovelo Fogaça, Mariana Cabral Schveitzer

**Affiliations:** 1 Universidade Federal de São Paulo, Grupo de Pesquisas CUIDAR-Promoção da Saúde e Práticas Integrativas e Complementares em Saúde, São Paulo, SP, Brazil.; 2 Universidade Federal de São Paulo, Escola Paulista de Medicina, Departamento de Medicina Preventiva, São Paulo, SP, Brazil.

**Keywords:** Occupational Health, Complementary Therapies, COVID-19, Primary Health Care, Health Promotion, Salud Laboral, Terapias Complementarias, COVID-19, Atención Primaria de Salud, Promoción de la Salud, Saúde do Trabalhador, Terapias Complementares, COVID-19, Atenção Primária à Saúde, Promoção da Saúde

## Abstract

**OBJECTIVE:**

To identify the possible repercussions of the COVID-19 pandemic on the workers’ health, the care strategies used, and the provision of Integrative and Complementary Practices in health services in the context of COVID-19.

**METHOD:**

Descriptive study, with a qualitative approach, which used a focus group for data collection and content analysis according to Bardin.

**RESULTS:**

Eleven health professionals from the city of Registro (SP) participated and, based on the information analysis, four categories emerged: (1) Changes in work routine caused by the pandemic and the feelings they generate in health professionals; (2) Integrative and Complementary Practices as a self-care strategy in the pandemic; (3) Provision of Integrative and Complementary Practices during the pandemic; and (4) Occupational health as the focus and strategy motivator to resume the provision of Integrative and Complementary Practices in the municipality.

**CONCLUSION:**

This study allowed the identification of the impact of the pandemic, especially on workers’ mental health, which influenced the search for care strategies that included the Integrative and Complementary Practices. Professionals with this training began to offer the Integrative and Complementary Practices in the service to other workers, given the interruption of their provision to the population due to the COVID-19 pandemic.

## RESUMO

**Objetivo:**

Identificar as possíveis repercussões da pandemia de COVID-19 na saúde dos trabalhadores, as estratégias de cuidado utilizadas e a oferta de Práticas Integrativas e Complementares em serviços de saúde no contexto da COVID-19.

**Método:**

Estudo descritivo, com abordagem qualitativa, que utilizou grupo focal para coleta de dados e análise de conteúdo segundo Bardin.

**Resultados:**

Participação de 11 profissionais de saúde da cidade de Registro (SP) e, a partir da análise de informações, emergiram quatro categorias: (1) As modificações na rotina de trabalho causadas pela pandemia e os sentimentos gerados nos profissionais de saúde; (2) Práticas Integrativas e Complementares como estratégia de autocuidado na pandemia; (3) A oferta de Práticas Integrativas e Complementares durante a pandemia; e (4) A saúde do trabalhador em foco e motivadora da estratégia para retomada da oferta de Práticas Integrativas e Complementares no município.

**Conclusão:**

Neste estudo, foi possível identificar o impacto da pandemia especialmente na saúde mental dos trabalhadores, o que influenciou a busca de estratégias de cuidado que incluíram as Práticas Integrativas e Complementares. Os profissionais com essa formação começaram a ofertar as Práticas Integrativas e Complementares no serviço aos demais trabalhadores, devido ao interrompimento da oferta das Práticas Integrativas e Complementares à população em consequência da pandemia de COVID-19.

## RESUMEN

**Objetivo:**

Identificar las posibles repercusiones de la pandemia de COVID-19 en la salud de los trabajadores, las estrategias de cuidado utilizadas y la oferta de prácticas integrativas y complementarias en servicios de salud en el contexto de COVID-19.

**Método:**

Estudio descriptivo, con abordaje cualitativo, que utilizó grupo focal para recolección de datos y análisis de contenido según Bardin.

**Resultados:**

Participación de 11 profesionales de salud de la ciudad de Registro (SP) y, a partir del análisis de informaciones, emergieron cuatro categorías: (1) Las modificaciones en la rutina de trabajo causadas por la pandemia y los sentimientos generados en los profesionales de salud; (2) Prácticas integrativas y complementarias como estrategia de autocuidado en la pandemia; (3) La oferta de prácticas integrativas y complementarias durante la pandemia; y (4) La salud del trabajador en foco y motivadora de la estrategia para el recomienzo de la oferta de prácticas integrativas y complementarias en el municipio.

**Conclusión:**

En este estudio, fue posible identificar el impacto de la pandemia especialmente en la salud mental de los trabajadores, que influyó la búsqueda de estratégias de cuidado que incluyeron las prácticas integrativas y complementarias. Los profesionales con esa formación comenzaron a ofertar las prácticas integrativas y complementarias en el servicio a los demás trabajadores, ya que hubo interrupción en la oferta de las prácticas integrativas y complementarias a la población debido a la pandemia de COVID-19.

## INTRODUCTION

Different problems, such as psychic suffering, anxiety disorder, sleep disorders, risk of contamination, illness, and death, affect health workers involved in dealing with the COVID-19 pandemic. Therefore, there are several strategies not only to provide assistance and protection to the health of these professionals^(1)^, but also to establish special conditions to work performance in the midst of the epidemic, in the attention to working hours, and in actions to reduce occupational stressors^(2)^ to preserve the workers’ physical and mental health.

Evidence-based practices and changes made to services are fundamental in combating the pandemic. However, as workers’ health is a cross-cutting issue, identifying, in the personal dimension, the way in which health workers are feeling and dealing with the impacts of the pandemic on their routine and health can help to promote actions and measures in this very difficult period. In 2020, a document^(3)^ published by the Brazilian Ministry of Health sought to summarize the main evidence on the benefits of Integrative and Complementary Practices (*PICS*) for workers’ health. For example, auriculotherapy, for work stress, and yoga, for Burnout syndrome, as well as meditation, contribute positively to mitigating specific health conditions.

To address fatigue and burnout in healthcare workers during the pandemic, an interdisciplinary team from the Intensive Care Unit recommended the inclusion of breathing exercises, biofeed- back, and mindfulness. These practices can be used to mitigate acute episodes of stress and anxiety, while telehealth services can be used to allow peer support and occupational counseling^(4)^.

Integrative and Complementary Practices were instituted through the National Policy on Integrative and Complementary Practices (*PNPIC*) of the Brazilian Public Health System (SUS), in May 2006^(5)^. Currently, 29 Integrative and Complementary Practices are available in SUS and are also present at all points of the Health Care Network (*RAS*), primarily in Primary Health Care (PHC). Some common points among the different practices included are the expanded view of the health-disease process, the promotion of self-care, and quality of life^(3,5,6)^.

Between February 2008 and July 2020, the information, which was extracted from the official database of the Department of Informatics of SUS (*DATASUS*) and generated by the TABNET application, indicated the provision of more than 11,772 procedures related to complementary therapies in Vale do Ribeira, in the southern region of the state of São Paulo, which includes the municipality of Registro, with emphasis on the practice of acupuncture, with 8,606 sessions carried out in this period^(6)^.

During the development of a project to promote health and complementary therapies as a care strategy for workers in the Vale do Ribeira area, it was decided that an online focus group would be formed by workers from the area covered by the Occupational Health Reference Center of Registro (CEREST-Registro)^(6)^. This way, it was possible to listen to perceptions and exchange experiences as part of the data collection strategy of a case study. The objectives of this study were to identify (a) the possible consequences of the pandemic on the workers’ health, (b) the care strategies used and (c) the provision of Integrative and Complementary Practices in health services in the midst of the COVID-19 pandemic.

## METHOD

### Design of Study

This is a descriptive study, with a qualitative approach, part of a case study^(7)^, which used the focus group technique^(8)^ for data collection and content analysis according to Bardin^(9)^.

### Population

Health services workers and managers from the Municipal Health Department (*SMS*) and of the Family Health Strategy teams (FHS).

### Local

The research scenario included the following organizations linked to Primary Care (PHC) health services in the municipality of Registro (SP): Municipal Health Department (*SMS*) and seven Family Health Strategy (FHS) Units, which belonged to four health districts (*DS*).

### Data Collection

The focus group is widely used in public health research, aiming to obtain data from a group of individuals purposely selected^(8)^. To recruit focus group participants, a formal invitation was sent to the *DS* coordinators, and this request was also extended to all health workers, especially those who provide Integrative and Complementary Practices, as well as those who have training in some complementary therapy.

Data collection was carried out in two focus groups, using the platform *Google Meet*, on October 1 and 8, 2020. The groups, conducted by a moderator (ECP), had the participation of two observers (LZF and MCS) to ensure the continuity of the activity in case there were problems with the internet connection. The groups had an average duration of 50 minutes and were recorded. The direction of the debate was guided by the following questions: “How do you feel about the pandemic?”, “How is your daily routine, your practice?”, “How are you dealing with the changes?”, “What are the complementary therapies provided in the services?” and “Is there a demand for complementary therapies in the services?”.

### Data Analysis and Treatment

The focus group sessions were transcribed (ECP) and submitted to content analysis according to Bardin, going through the steps of (i) pre-analysis, (ii) exploration of the material, (iii) treatment of the results, and (iv) interpretation^(9)^. The identification of extracts and the elaboration of the categories were carried out in groups by all the authors. For the presentation of the results, the following codes were used for the participants: “P” for workers (*profissional* in Portuguese) or “G” for managers (*gestores*, in Portuguese); for services: “*SMS*”, “FHS” or “*DS*”; and, for the focus group session: GF-S “1” or “2”.

### Ethical Aspects

This project was approved by the Research Ethics Committee (CEP) of the Universidade Federal de São Paulo (UNIFESP), under number 3.802.064, in 2020. The individuals interviewed signed the Free and Informed Consent Form (FICF), in accordance with Resolution No. 466/12.

The focus group was part of a project funded by CEREST-Registro, and supported by the Municipal Health Department of Registro^(6)^.

## RESULTS

Eleven health workers participated in the focus groups, and the sessions were held in October 2020, with eight FHS professionals, two *DS* managers, and one *SMS* manager. Of these, ten were women and one, a man. As for education, nine participants had an undergraduate degree in Nursing, one participant had a degree in Physiotherapy, and one was a community health agent. Five participants had training in the following complementary therapies that make up the PNPIC: acupuncture, auriculotherapy, and flower therapy.

The group discussions provided an in-depth investigation of the provision of complementary therapies in Primary Care (PHC), exchange of experiences about workers’ health and their work process in the current pandemic scenario, and discussion of strategies for the challenges presented. From these discussions, four categories were elaborated, and the main excerpts are presented below:

### Category 1 – Changes in the Work Routine due to the Pandemic and Feelings Generated in Health Workers

The COVID-19 pandemic has considerably transformed the professionals’ work routine, requiring a new posture. Distancing from the family network, greater workload, difficulty in raising awareness among the population, and insertion of new protocols in the routine made the moment challenging and stressful, and led the workers to feel pressed, fearful, and insecure.

*(…) it is something that we are tired of already and would like it to end soon. Like, the feeling is: ‘it’s over’ and it isn’t, you know, and we need to find more strength to hold on a little longer because it’s not over yet, right? I think the most distressing thing, in my situation, is this issue of the family, of not having contact with them, anyway, that’s it.* (G1/DS GF-S1)

*Daily routine has been very disturbed. I don’t know if I can use that word, much more agitated than we were used to, you know, with actions being integrated, like, in our daily lives, in a very crazy way and that, sometimes, we are not even prepared to that.* (P1/FHS GF-S1)

*So, I think that, in the beginning, for everyone it was a mixture of feelings, right? It’s fear, insecurity, so sometimes we don’t know how to act in the face of something unknown, in the face of changes in protocol, in front of our family, in front of the team, and everyone expects a lot from you.* (P2/FHS GF-S1)

*So, this moment, it’s been a very challenging period, with a very important workload there, right, that we’re trying to balance, right, between the demands that we already carried, you know, as our responsibility and, adding to this demand, this situation, right, the pandemic.* (P3/FHS GF-S2)

*It’s pretty stressful. Actually, it’s day by day, and trying to get adapted to the conditions we’re having at the moment.* (P5/FHS GF-S2)

### Category 2 – Integrative and Complementary Practices as a Self-Care Strategy in the Pandemic

Self-care is essential for health maintenance and preservation and, in the context of the pandemic, its importance for the population as a whole became more present, which was no different for the participants of this research. Reiki, meditation, aromatherapy, and flower therapies were the practices mentioned as self-care strategies used.

*(…) I have been trying to meditate a little to help, the self- application of Reiki, too, has helped me a little (…)* (P1/FHS GF-S1)

*Aromatherapy (…) meditation, too, I have used it at home.* (G2/SMS GF-S1)

*(…) at home I like to meditate (…).* (P4/FHS GF-S2)

*I try to meditate (…).* (P5/FHS GF-S2)

*(…) since before the pandemic, I already had the meditation process for self-care (…).* (P6/FHS GF-S2)

*(…) at home, too, meditation (…) I also use a lot of essential oil, incense, I work a lot on this issue of aromatherapy at home.* (G3/DS GF-S2)

*So, in my care, I use quantum flower remedies (…).* (P7/FHS GF-S2)

### Category 3 – Integrative and Complementary Practices Provision During the Pandemic

Based on the participants’ reports, it was possible to identify different dynamics in the offer of complementary therapies during the pandemic:

- interruption of the supply of complementary therapies to the population and search for strategies for its return;
- interruption of the provision of complementary therapies to the population and its orientation to co-workers, especially auriculotherapy;
- provision of complementary therapies, initially, directed to co-workers to, later, be offered to the general population;
- introduction of complementary therapies with greater ease in the workers’ routine of care; and
- referral of cases to a reference professional.

*Here at the unit, I’m the only one to practise, you know. The other colleagues here in this unit did not have these practices. And, for some, I have started to make auriculotherapy, who came with this need. Slowly, I’m trying to introduce here to the unit first with my co-workers.* (P1/FHS GF-S1)

*(…) So, I even talked to my doctor here (…) she will end up referring some patients that she observes, who need this complementation, so that we can start introducing them again. (…) I have done some, not as routinely as it should be, but I have done (auriculotherapy) on some people in the team (…).* (P2/FHS GF-S1)

*Here I don’t know how to do it, so I don’t do it, but I have already referred patients to the Naturopathic Doctor that we have in Registro, and I have referred the case to him.* (P4/FHS GF-S2)

*Well, here at the unit, what I can offer in a more frequent routine, there are even some patients I saw weekly, is auriculotherapy. This is a service that I can provide and there are some patients that I can do acupuncture myself, but these are patients… that I do not have time to do acupuncture here in the unit, but is done more routinely, well, it’s auriculotherapy too, and the cupping.* (P5/FHS GF-S2)

*(…) we had everything programmed to start the consultations (auriculotherapy) in the units, but then the pandemic came and it was suspended. And then I, on my own, attended, at the unit, the employees who accepted.* (P6/ FHS GF-S2)

*Here in our unit, I can also attend to auriculotherapy, right, to include it in our activity and the cupping therapy, right, too, when needed. The other techniques that demand more, it gets a little delicate, you know (…) And then the patients are waiting for me to return to routine care so that I can return with them, you know. So, right now, I’m supporting my colleagues here, that’s all.* (P7/FHS GF-S2)

### Category 4 – Worker’s Health in Focus and as a Strategy for Resuming the Provision of Integrative and Complementary Practices in the Municipality

In addition to modifying the work process at PHC, the COVID-19 pandemic shows the need to prioritize the workers’ health. Participants understand that, at first, resuming or starting to care with complementary therapies for health workers would be the best option to reorganize its provision in the municipality.

*I think it’s cool. I think it’s even a way for us to organize these services and then open them to the population, right? (…) In addition to taking care of those who take care, right, because it’s very complicated, sometimes we demand so much from our colleagues here, employees, but they are sometimes sicker than the population, you know. A way to value them too.* (P1/FHS GF-S1)

*I think the team comes. It has to come first, because a team that feels welcomed, that feels that we professionals are paying attention to it, they can perform much better. (…) Put it as something scheduled, do all that conversation that is necessary, you know, and do all that follow-up. I think it’s a very good proposal and I’m going to implement it here.* (P2/FHS GF-S1)

*But I believe that we should, at this first moment, think about caring for the worker, about the team self-care, so that, later, when this is all very organized, structured, offer it to the population in its entire context.* (G2/SMS GF-S1)

*I, in particular, think it would be great if it started with the health workers because we are the direct caregivers of the community in each territory, so if we are strengthened, we have more to contribute to the community. Then spread and multiply, you know I think it would be wonderful.* (P3/FHS GF-S2)

*I agree too. I think it will be very good and it will improve the work process a lot, of taking care of the workers first. Even for us to be able to take care of the other, we need to take care of ourselves, right?* (P4/FHS GF-S2)

*So, I think it’s important to start with the worker because, well, we’re in a difficult time, because of the pandemic we’re going through, which, like it or not, is very stressful, it’s a new thing. You have to be reinventing yourself, getting adapted every day. For another matter, too, this year is an election year. It’s another stress for us too. So, yes, I think it’s time to think about the worker indeed.* (P5/FHS GF-S2)

*Not only for health workers, but for all public servants, right, in the municipality of Registro, but starting with the health worker to support us because then we would also be benefited, right, in this environment.* (P7/FHS GF-S2)

*I think it’s interesting, because workers in general, right, in health, we really need it, you see? This view is very interesting. (…) the Naturopathic Doctor, he is already doing this, opening his schedule to assist the workers and, just as he helped me, he can help other people as well.* (P8/FHS GF-S2)

## DISCUSSION

Among the integrative and complementary practices that make up the *PNPIC*, the participants mentioned training in acupuncture, auriculotherapy, and flower therapy. The first two complementary therapies are part of the integral system of Traditional Chinese Medicine for health promotion and disease treatment. The first one uses the insertion of needles into acupuncture points; and the second places seeds or metallic spheres in points on the pinna. The third one, flower therapy, is a therapeutic practice that uses flower essences to balance and harmonize the individual^(6)^.

A project funded by the Brazilian Ministry of Health (*MS*), in partnership with the Brazilian Academic Consortium for Integrative Health (*CABSIN*) and the Regional Library of Medicine/ Pan American Health Organization/World Health Organization (BIREME/PAHO/WHO), sought to systematize, through maps, scientific evidence on Traditional, Complementary, and Integrative Medicines (TCIM) to support health workers, decision makers, and researchers in the construction of evidence-based health actions. These maps present an overview of the evidence in complementary therapies for specific health problems, including a map on acupuncture and a map on auriculotherapy, both of which are available on the *site* of the Virtual Health Library (VHL) in MTCI (https://mtci.bvsalud.org/pt/mapas-de-evidencia-2/).

When analyzing the 30 years of *SUS* ^(5)^, Integrative and Complementary Practices stands out as an important strategy in the redirection of the care model, as well as in the improvement of care integrality and the expansion of therapeutic options, in the face of a scenario of demographic-epidemiological transition. Specifically, complementary therapies from traditional knowledge are included in the field of primary care, encouraging the use of available cultural and community resources, contributing to the production of changes in care and in the health services daily routine.

The pandemic produced new challenges for professionals, for example, dealing not only with the work routine directed to the new demands of the current context, but also with the emergence of feelings that can affect mental health. To minimize the negative effects of the pandemic, professionals use complementary therapies both for self-care and for the health care of co-workers. The reorganization of the provision of complementary therapies, aiming to improve the attention to the worker’s health, can be a strategy to maintain and/or resume the provision of these practices in the municipality.

PHC is the gateway and the organizer of care in the complex health care network of SUS^(10)^. The professionals working there gain greater prominence in solving the health demands of the territories and are more exposed to risks. In addition to vulnerability to biological risks, such as contamination by the virus, the COVID-19 pandemic highlights the psychological suffering to which these professionals are exposed in the work environment. A review evaluated the relationship between factors linked to the workplace and the workers’ mental health during the COVID-19 pandemic, revealing that these workers are more likely to have increased symptoms of depression, stress, anxiety, insomnia, among others^(11)^.

Participants in this study reported a heavy workload, distancing from the socio-affective network, unpleasant feelings and sensations in relation to changes in the work routine and the management of their own relationships. These issues are expected in the current context of a pandemic and directly reflect on the quality of life and self-care of these professionals. Therefore, the adoption of mental health care strategies for these workers is important and, thus, ensure their well-being to perform their duties^(12)^. Accurate updating of information about the disease, training in the use of personal protective equipment, monitoring of the team well-being, and mapping and dissemination of actions regarding the care available to workers should be measures to be incorporated in the recommendations to managers^(12)^.

This study showed that complementary therapies are adopted as a self-care strategy in the pandemic by workers who have training in one of the practices. Among the practices mentioned, meditation presents robust clinical evidence in mental health care. The mapping of possible evidence of complementary therapies in the context of COVID-19 shows that meditation techniques have “positive” and “potentially positive” effects for the management of post-traumatic stress, anxiety, depression, sleep disorders, work and psychological stress, among others. The studies included in the mapping and that investigated this practice highlight evidence with a confidence level of “moderate” to “high”^(13,14)^.

The participants’ reports and the protection measures that were imposed in the municipality allowed the observation of a reduction in the supply of complementary therapies to the population of the municipality of Registro (SP) due to the needs of adaptation in assistance in the context of the pandemic. In this scenario, the provision of complementary therapies was directed to meeting the demand that arose from co-workers.

In 2020, there were initiatives to offer complementary therapies due to the pandemic, and the actions of the Nursing Care Network (https://redecuidarenfermagem.com.br/) and the Integrative and Complementary Practices Collaborative Network (https://www.ufrgs.br/levi/rede-colaborativa-*PICS*/#page-content) stood out in occupational health care. These collaborative projects received support from the Federal Nursing Council (*CFE*) and the National Health Council (*CNS*), respectively, and had the participation of professionals from different backgrounds, SUS workers and volunteer therapists, who offered several complementary therapies through individual or collective teleservices.

The provision of complementary therapies in PHC increased by 324% between 2017 and 2019, especially the practice of auriculotherapy, with an increase from 40,818 to 423,774 records^(15)^. This amount can be explained by the offer of training in this practice to PHC health professionals, which began in 2016. The agreement between the National Coordination of Integrative Practices and the Universidade Federal de Santa Catarina has already trained more than 10,000 higher education professionals^(16)^. This practice already has evidence-based clinical recommendations for the treatment of smoking, anxiety, insomnia, obesity and low back pain, which are prevalent conditions in PHC, and these protocols can be directed to the workers’ health.

Studies indicate that the use of auriculotherapy in health professionals during the pandemic contributed to minimize pain; reduce symptoms of stress, depression and anxiety; promote the strengthening of bonds; and also improve the work environment^(17,18)^. This is one of the practices the participants of this study used in their co-workers health care. Auriculotherapy and other therapies, such as acupuncture, as well as practices related to the mind and body of Traditional Chinese Medicine, such as *Tai Chi Chuan and Qi Gong*, gather clinical evidence for pain conditions, treatment of several chronic and acute diseases, mental health and quality of life^(19,20,21,22)^.

The WHO has designated 2021 as the International Year of Health Workers and Caregivers as a way of recognizing the dedication of healthcare professionals, responsible for the front line care of COVID-19. Thus, complementary therapies reveal themselves as a powerful health care strategy for these workers, as they address physical, emotional, social and also spiritual aspects of health care^(23)^.

The continuity of supply of complementary therapies during the pandemic at the initiative of some of the workers participating in the research demonstrates this care, but the recognition of managers and the proper registration is fundamental for the expansion of these practices at *SUS*. In addition, even though the *PNPIC* has existed for 15 years, the mobilization of institutional resources for its maintenance and expansion is increasingly necessary^(24)^.

The focus group, therefore, allowed an exchange of experiences and care practices in this complex moment of the pandemic, including complementary therapies. However, this exchange could have been enhanced if the group sessions had taken place in person. This, in fact, was the initial proposal of the project. It can be inferred, therefore, that the online format of the focus group was one of the limitations of this study, since some people quit and/or abandoned the sessions due to technological difficulties or lack of contact, which, in its turn, can reduce the idea of group belonging.

## CONCLUSION

This study allowed the identification of the impact of the pandemic on workers’ health, especially on mental health. This situation influenced the search for care strategies that included complementary therapies, as the professionals with adequate training began to offer these practices in the service to other workers, given the interruption of their provision to the population due to the COVID-19 pandemic.

Countless were – and continue to be – the challenges in the routine of health workers during the pandemic. In this study, complementary therapies were cited as a self-care strategy, and their provision to workers during the pandemic period revived the discussion about the importance of occupational health. The workers even indicated the following strategy for the resumption, at PHC, of complementary therapies, which were interrupted in the second half of 2020 due to the pandemic: first the practices should be offered to workers and, later, to the entire population.

This way, besides adapting services to ensure health and safety for workers during the pandemic, the construction of support networks and the systematization of the provision of complementary therapies to workers during the workday can help mental health care and health promotion.

## ASSOCIATE EDITOR

Cássia Baldini Soares
